# Supplementary material for: Quiescent cells maintain active degradation-mediated protein quality control requiring proteasome, autophagy, and nucleus-vacuole junctions
Source: J Biol Chem. 2024 Nov 29;301(1):108045. doi: 10.1016/j.jbc.2024.108045 (PMC11731230; doi:10.1016/j.jbc.2024.108045)
Supplement: Supporting information Figures S1–S9 [file mmc1.pdf]

**Quiescent cells maintain active degradation-mediated protein quality control requiring proteasome, autophagy and nucleus-vacuole junctions**

Dina Franić, Mihaela Pravica, Klara Zubčić, Shawna Miles, Antonio Bedalov, Mirta Boban*

## Supporting information

## Figure S1 – S9

## Supplementary Figure S1

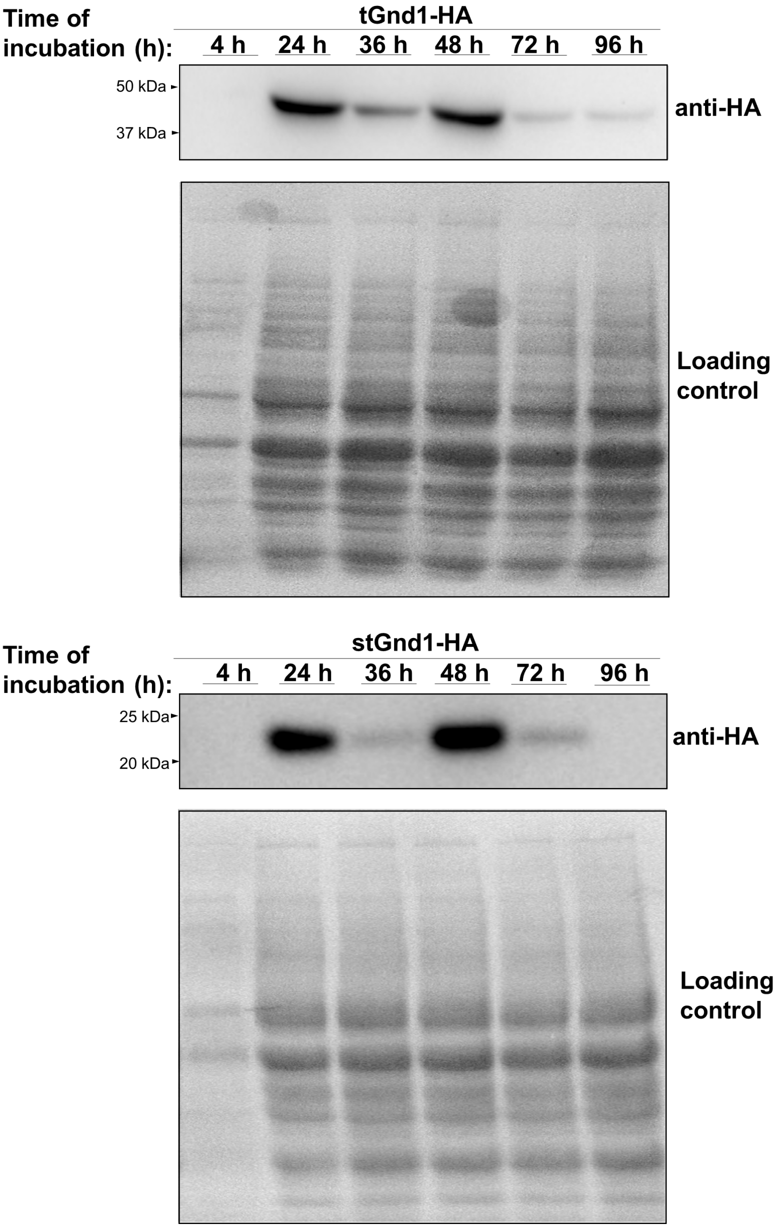


**Figure S1. Expression of model misfolded proteins tGnd1-HA and stGnd1-HA under the control of constitutive *PIR3*-gene promoter in proliferating and quiescent cells.** Western blot analysis of tGnd1-HA (DFY001) and stGnd1 (DFY002) expressed under the control of *PIR3*- promoter in the wild type BY4741 strain. Cells were inoculated at an initial optical density ($OD_{600}$) of 0.2 and cultured for an indicated time period. Cell lysates were analyzed by Western blot (anti-HA). Stain-free total protein (Bio-Rad) was used as a loading control.

## Supplementary Figure S2

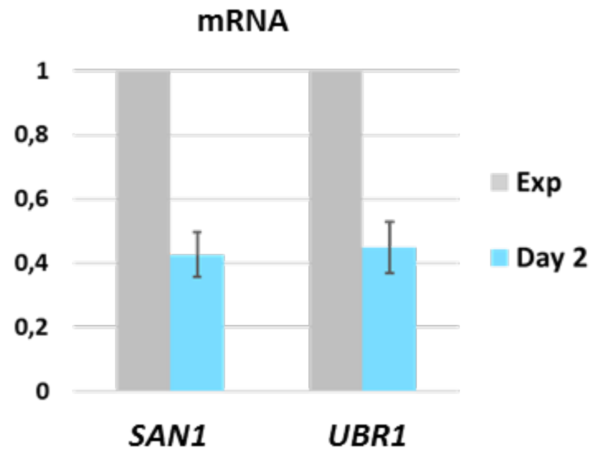


**Figure S2. mRNA levels of *SAN1* and *UBR1* in proliferating and quiescent cells.** *SAN1* and *UBR1* mRNA levels were analyzed in cells from exponentially growing cultures (strains DFY043 and DFY039, respectively), and in cells from two days old cultures (strains DFY041 and DFY037, respectively). Mean values of $2^{-\Delta\Delta CT}$ from two biological replicates and standard deviation are shown.

## Supplementary Figure S3

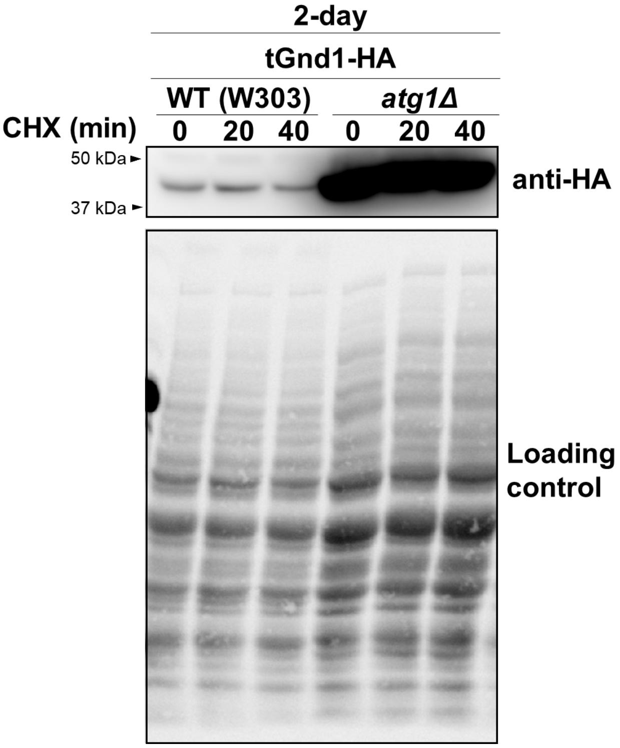


**Figure S3. Misfolded protein tGnd1-HA is stabilized in quiescent cells of the *atg1Δ* mutant of the W303 strain background.** Western blot analysis of cycloheximide chase (performed as in Fig.1). The stability of tGnd1-HA in wild-type (MPY166) and *atg1Δ* mutant (MPY170) cells from 2 days old cultures was analyzed. Stain-free total protein (Bio-Rad) was used as a loading control.

## Supplementary Figure S4

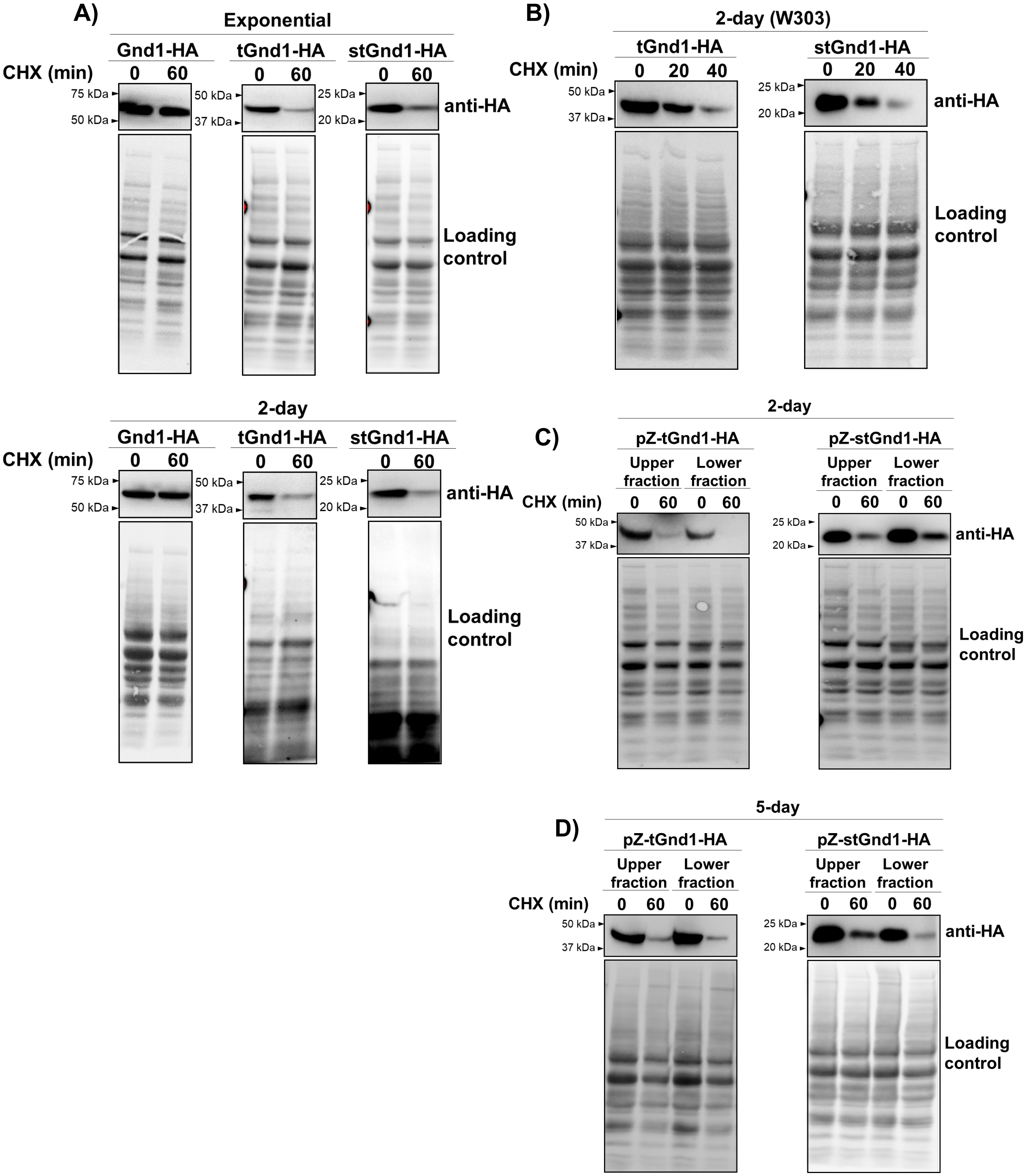


**Figure S4. Related to main Fig. 1 B-E.** Full lanes of stain-free total protein (Bio-Rad) are shown for each Western blot from the main Fig. 1 B-E.

## Supplementary Figure S5

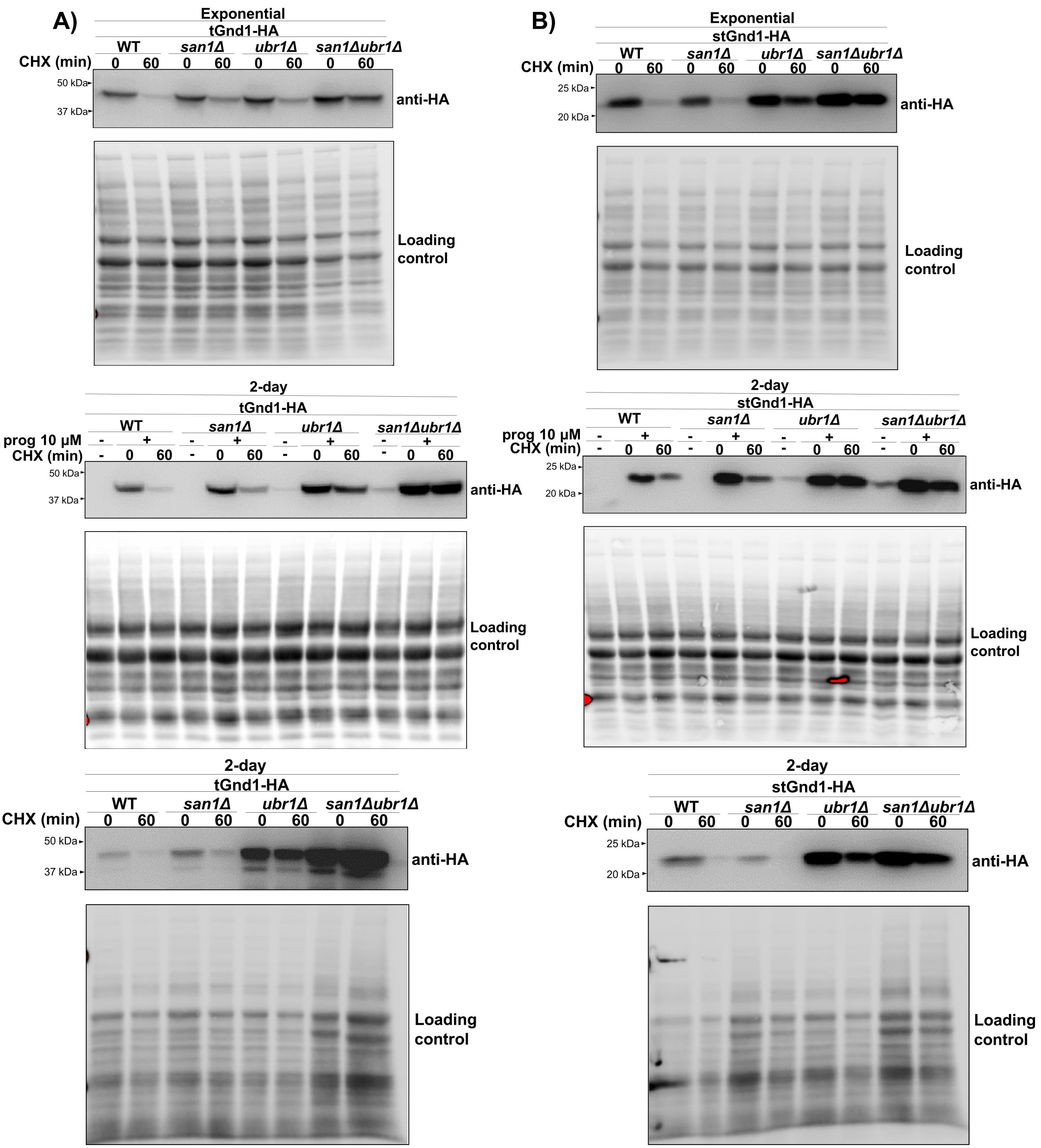


**Figure S5. Related to main Fig. 2 A-B.** Full lanes of stain-free total protein (Bio-Rad) are shown for each Western blot from the main Fig. 2 A-B.

## Supplementary Figure S6

A)

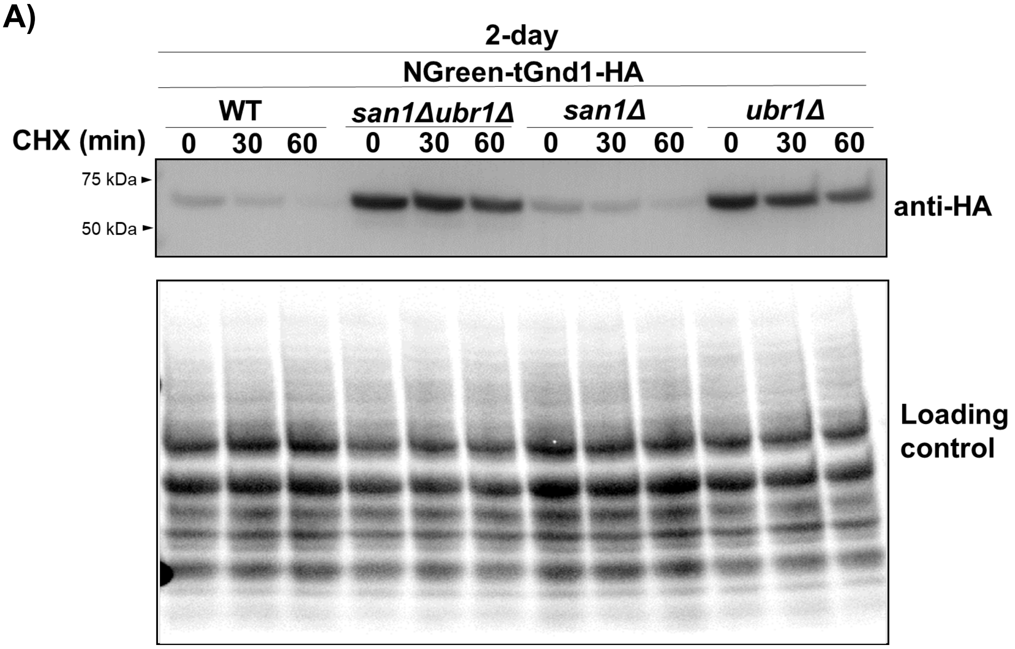


B)

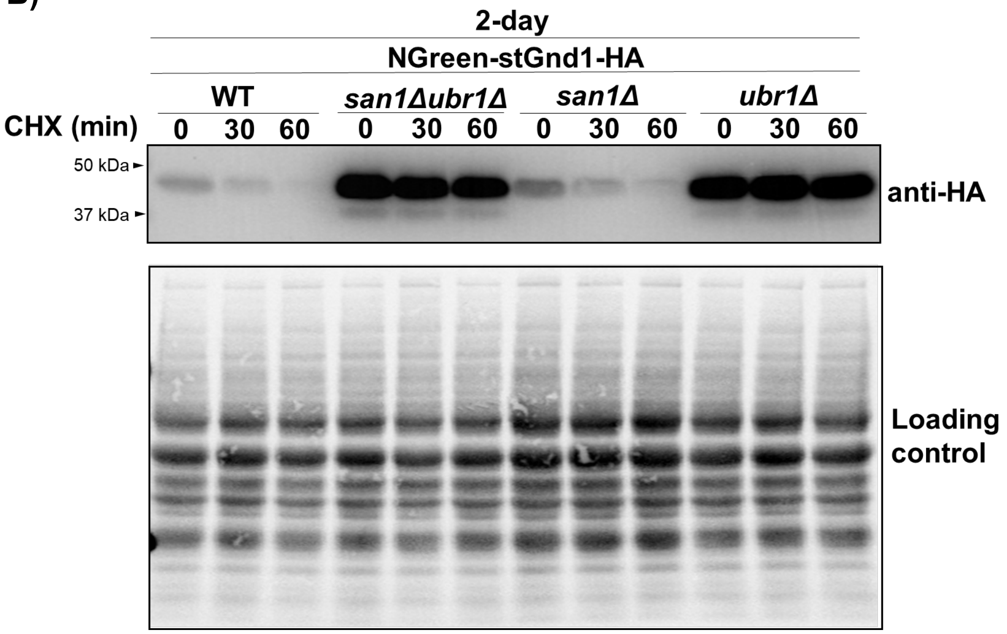


**Figure S6. Related to main Fig.3 A-B.** Full lanes of stain-free total protein (Bio-Rad) are shown for each Western blot from the main Fig. 3 A-B.

## Supplementary Figure S7

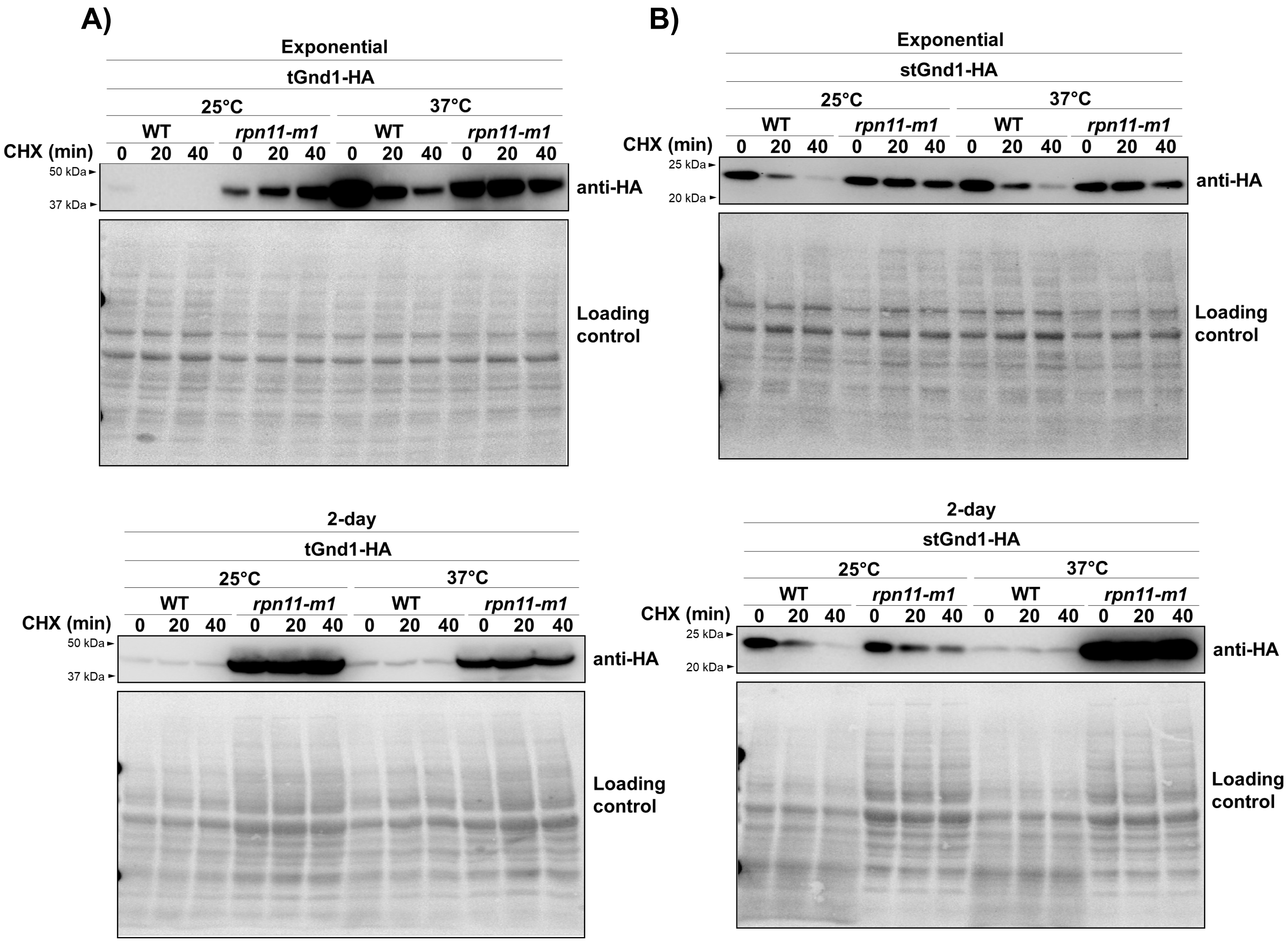


**Figure S7. Related to main Fig.4 A-B.** Full lanes of stain-free total protein (Bio-Rad) are shown for each Western blot from the main Fig. 4 A-B.

## Supplementary Figure S8

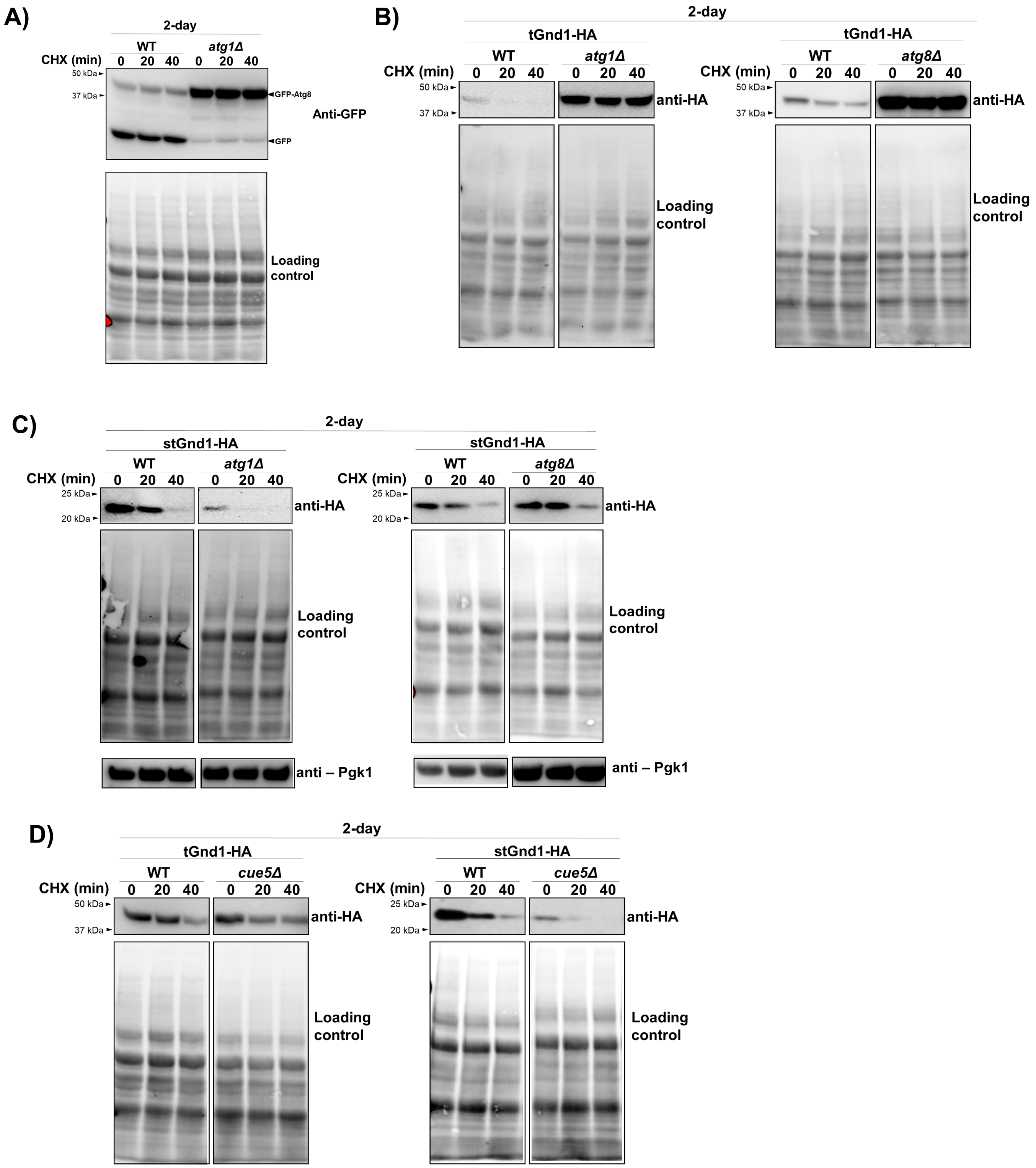


**Figure S8. Related to main Fig.5 A-D.** Full lanes of stain-free total protein (Bio-Rad) are shown for each Western blot from the main Fig. 5 A-D.

## Supplementary Figure S9

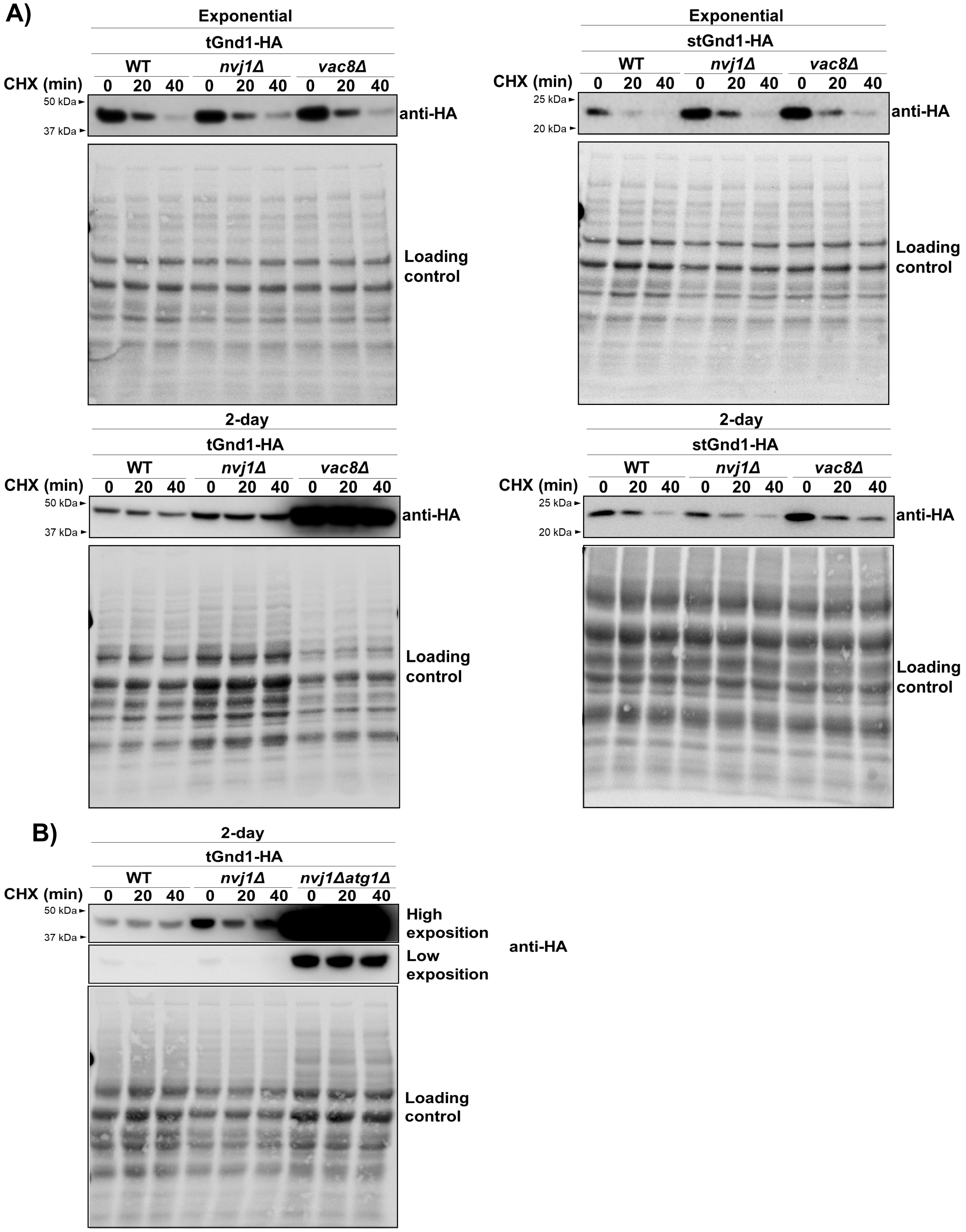


**Figure S9. Related to main Fig.6 A-B.** Full lanes of stain-free total protein (Bio-Rad) are shown for each Western blot from the main Fig. 6 A-B.
